# Multimodal optical imaging strategy to evaluate the microscopic biodistribution of SERS gold nanoparticles across histology sections

**DOI:** 10.1117/1.BIOS.3.4.043104

**Published:** 2026-09-10

**Authors:** Boyi Wang, Alexander Czaja, Olga E. Eremina, Sean Burkitt, Pegah Bagheri, Helen R. Salinas, Fernando Largaespada, Cristina Zavaleta

**Affiliations:** a University of Southern California, Department of Biomedical Engineering, Los Angeles, California, United States; b University of Southern California, USC Michelson Center for Convergent Bioscience, Los Angeles, California, United States

**Keywords:** Raman spectroscopy, multiphoton luminescence, surface-enhanced Raman scattering nanoparticles, multimodal imaging, biodistribution

## Abstract

**Significance:**

Surface-enhanced Raman scattering (SERS) gold nanoparticles (AuNPs) are widely used for molecular imaging and diagnostics, but their non-biodegradable nature necessitates accurate, spatially resolved methods to assess long-term biodistribution. Existing approaches often lack sensitivity and spatial resolution or require destructive sample preparation.

**Aim:**

We evaluate multiphoton luminescence (MPL) microscopy as a label-free approach for detecting SERS AuNPs in biological tissues while using Raman imaging for spectroscopic validation of nanoparticle (NP) localization.

**Approach:**

SERS AuNPs were intravenously administered in mice at multiple doses and analyzed at 1- and 8-week time points using MPL and Raman microscopy. Quantitative analyses were used to compare NP signals across organs, doses, and time points.

**Results:**

MPL and Raman imaging both detected SERS AuNP-associated signals across all doses, with strong spatial correlation observed among modalities. Dose-dependent increases in signal were observed for both modalities, with normalized positive pixel area fraction in liver increasing from 0.02 at 0.1 nM to 0.99 at 5 nM. Whole-organ mapping revealed preferential accumulation in the liver and spleen, while longitudinal analysis demonstrated increased NP aggregation at later time points.

**Conclusions:**

MPL enables sensitive, label-free detection of SERS AuNPs and, when combined with Raman imaging, provides a non-destructive framework for spatially resolved biodistribution analysis.

Statement of Discovery Multiphoton luminescence (MPL) microscopy enables label-free detection of surface-enhanced Raman scattering gold nanoparticles in biological histology tissues, with Raman imaging providing spectroscopic validation of nanoparticle (NP) identity. We demonstrate strong spatial overlap and quantitative agreement between MPL and Raman signals across doses, organs, and time points. This multimodal optical imaging approach enables non-destructive, spatially resolved assessment of long-term NP biodistribution and aggregation.

## 1 Introduction

Gold nanoparticles (AuNPs) are widely explored and used in the biomedical field due to their unique optical, chemical, and physicochemical properties, enabling applications ranging from molecular imaging and diagnostics to drug delivery and cancer therapy.^1^^–^^6^ Among the various AuNPs developed, surface-enhanced Raman scattering (SERS) AuNPs have the potential to revolutionize cancer diagnostics and therapy by enabling enhanced imaging and precise molecular detection at the cellular level.^7^^–^^9^ They stand out with their high sensitivity due to their ability to amplify inelastic light scattering signals through surface plasmon resonance (SPR), allowing detection at extremely low concentrations.^10^ Importantly, these nanoparticles (NPs) can be engineered with tunable properties and alternative Raman reporter layers responsible for eliciting distinct spectral fingerprints, allowing for unprecedented levels of multiplexing.^11^^–^^13^ They have also demonstrated a high degree of passive accumulation in tumors post-intravenous (IV) injection with the potential to enable tumor localization and guide surgical resection.^14^^–^^16^ However, SERS AuNPs are inherently non-biodegradable due to their gold core and silica-based encapsulation.^17^^,^^18^ This raises concerns regarding their long-term retention in the body and potential toxicity, highlighting the urgent need to better understand the biodistribution and fate of these NPs *in vivo*, particularly over extended time scales.

Conventional strategies for evaluating AuNP biodistribution rely on a combination of optical imaging and analytical techniques. Brightfield microscopy is frequently used in histological analysis to assess tissue morphology.^19^^,^^20^ Samples typically undergo standard preparation workflows, including fixation, paraffin embedding or cryosectioning, and staining such as hematoxylin and eosin (H&E).^21^^,^^22^ Although these steps provide valuable contextual information on tissue architecture and pathology, brightfield imaging offers limited intrinsic contrast for AuNPs and lacks the specificity required to reliably distinguish nanoparticle signal from endogenous tissue features, including pigments, dense cellular structures, or staining artifacts.^23^^,^^24^ As a result, individual NPs or small NP aggregates cannot be directly visualized using brightfield microscopy alone. Fluorescence microscopy is commonly used to visualize AuNP distribution at the cellular level, but it typically depends on externally conjugated fluorophores.^25^^,^^26^ These labels are susceptible to photobleaching, chemical degradation, and signal quenching in biological environments and may dissociate from the NP surface over time, leading to discrepancies between the fluorescent signal and true NP localization.^27^^–^^30^ Inductively coupled plasma mass spectrometry (ICP-MS) is also widely used to quantify gold content within excised tissues. Although ICP-MS offers high sensitivity and quantitative accuracy, it requires complete tissue digestion and therefore eliminates all spatial information.^31^^,^^32^ As a result, this approach cannot resolve heterogeneous NP distribution within organs or distinguish among vascular, interstitial, and intracellular localizations. Radiolabeling approaches, such as positron emission tomography and single-photon emission computed tomography, enable sensitive whole-body tracking of NPs for systemic biodistribution studies.^33^^–^^35^ However, these methods offer limited tissue and cellular-level resolution and rely on radioactive isotopes that may detach from the NP surface or undergo decay, reducing their reliability for long-term biodistribution analysis.^36^^,^^37^ High-resolution electron microscopy techniques, including transmission electron microscopy and scanning electron microscopy (SEM), enable direct visualization of AuNPs without the need for external labels and provide nanometer-scale structural detail.^38^^–^^41^ However, their application is limited by small fields of view, extensive sample preparation requirements, and low throughput, making them impractical for whole-organ or longitudinal biodistribution studies.^19^^,^^42^

We recently developed a new label-free imaging approach that addresses the mentioned problems by utilizing multiphoton luminescence (MPL) to identify and localize AuNPs in animal models post-IV injection.^24^ Under excitation by ultrafast near-infrared femtosecond laser pulses, AuNPs generate broadband luminescence through nonlinear electronic processes that are strongly enhanced by localized SPR.^43^ The nonlinear excitation mechanism confines signal generation to the focal volume, providing intrinsic optical sectioning and high spatial resolution with reduced background autofluorescence.^44^^–^^48^ Because the MPL signal originates directly from the metallic core, it enables sensitive NP detection without external dyes, fluorophores, or reporter molecules.^43^^,^^44^ These properties make MPL well suited for imaging AuNPs in biological tissues and for repeated or long-term biodistribution studies.

Raman microscopy provides a valuable spectroscopic approach for studying SERS AuNPs through the detection of characteristic vibrational fingerprints associated with the NP-bound reporter molecules.^8^^,^^28^^,^^29^^,^^49^ The unique spectral fingerprint of each SERS NP provides an unmistakable signature, ensuring that its presence within biological tissues can be definitively confirmed without interference from background signals.^10^^,^^50^ Raman-based techniques have also been extended to *in vivo* applications through spatially offset Raman spectroscopy (SESORS), which enables recovery of Raman signals from subsurface regions by collecting photons at spatial offsets from the excitation site.^51^^,^^52^ By decoupling the excitation and collection geometries, SESORS increases sensitivity to deeper-lying Raman signals and has enabled non-invasive detection of SERS AuNPs through >40  mm of biological tissue.^53^^–^^55^ These advances have supported a growing number of *in vivo* biodistribution studies using SERS AuNPs for tumor detection, image-guided surgery, and molecular profiling.

Current approaches for assessing NP biodistribution remain limited by an inability to provide non-destructive, spatially resolved characterization over extended time scales, hindering efforts to understand the long-term fate of SERS AuNPs *in vivo*. In this study, we establish a multimodal imaging framework that leverages MPL for label-free visualization of AuNP distribution and Raman microscopy for spectroscopic validation of SERS AuNP identity. SERS AuNPs were IV-administered in murine models at 0.1, 1, and 5 nM and evaluated at 1- and 8-week time points. Whole-organ imaging and region-of-interest (ROI) analyses were performed to assess NP localization and aggregation behavior. Signal overlaps between MPL and Raman signals demonstrated that regions identified by MPL correspond to Raman-confirmed SERS AuNP signals. Both MPL and Raman imaging successfully detected SERS AuNP-associated signals across all doses. Results showed a clear dose-dependent increase in signal for both modalities, with strong correlation observed between MPL and Raman measurements across organs and experimental conditions. Whole-organ mapping demonstrated preferential accumulation of NPs in the liver and spleen, with comparatively lower signal in the lung, kidney, and brain. Longitudinal analysis further revealed time-dependent changes in NP distribution, including a shift toward larger aggregate sizes at 8 weeks relative to 1 week. By integrating these optical modalities, this research establishes a robust approach for characterizing the long-term fate and tissue-level distribution of SERS AuNPs in biological systems, paving the path for future biodistribution studies using our multimodal, label-free imaging approach.

## 2 Materials and Methods

### 2.1 SERS AuNP Fabrication and Characterization

AuNPs were synthesized using a hydroxylamine-assisted seed growth method conducted at low temperature. Briefly, 6.0 mL of a 30  mg/mL gold(III) chloride hydrate solution (${\mathrm{HAuCl}}_{4}\cdot xH_{2}O$, 99.995%, Sigma-Aldrich, St. Louis, Missouri, United States) was added to 900 mL of 4°C deionized water (Milli-Q grade, 18.2 MΩ·cm, Millipore, Burlington, Massachusetts, United States) under vigorous stirring, followed by rapid addition of 1.2 mL of a premixed 0.135  g/mL sodium citrate ($C_{6}H_{5}{\mathrm{Na}}_{3}O_{7}\cdot 2H_{2}O$, 99.0%, Sigma-Aldrich) and 0.085 g/mL hydroxylamine hydrochloride (98.0%, Sigma-Aldrich) solution. After 10 s, 2.1 mL of freshly prepared 0.0001% (w/v) sodium borohydride (${\mathrm{NaBH}}_{4}$, 98.0%, Sigma-Aldrich) in 1.0 M sodium hydroxide (NaOH, Sigma-Aldrich) was injected. The reaction was stirred for an additional 30 min in the cold room to yield a stable AuNP colloid. Raman labeling was performed using 100 mL of the as-prepared AuNP suspension diluted to 50 pM. Surface functionalization was achieved by dropwise addition of 100  μL of 0.1 mM (3-aminopropyl)trimethoxysilane (APTMS, 98%, Sigma-Aldrich) prepared in anhydrous ethanol (Acros Organics, Geel, Belgium), followed by incubation for 12 min under stirring. A Raman reporter, 1,2-bis(4-pyridyl)ethylene (BPE, 98%, TCI; 0.1 mM in ethanol, TCI, Tokyo, Japan), was then introduced dropwise, and the mixture was stirred for 5 min. The reaction was passivated by addition of 1.0 mL of 2.16 wt% sodium silicate solution (26.5% ${\mathrm{SiO}}_{2}$, Sigma-Aldrich), stirred for 30 min, and allowed to rest overnight. Silica encapsulation was carried out via the Stöber process by transferring the NPs into an ethanol-rich medium, followed by sequential addition of 2.5 mL ammonium hydroxide (${\mathrm{NH}}_{4}\mathrm{OH}$, 28 to 30%, Sigma-Aldrich) and 225  μL tetraethyl orthosilicate (TEOS, 99%, Sigma-Aldrich) to grow an ∼30  nm silica shell. The reaction proceeded under stirring at room temperature (RT) for 48 h. The SERS NPs were purified by centrifugation at 1500 g for 2 h, repeated five times. The final SERS NPs were stored at RT in the dark.

Successful Raman labeling of the SERS AuNPs was confirmed by acquiring Raman spectra using an RA816 Biological Analyser Raman microscope (Renishaw plc, Gloucestershire, United Kingdom). Measurements were performed using a 40× coverslip-corrected objective lens with a numerical aperture of 0.50 and a near-infrared diode laser operating at 785 nm, with laser power attenuated using neutral density filtering. NP concentration was determined by UV-Vis spectroscopy using a NanoDrop 2000 spectrophotometer (Thermo Scientific, Waltham, Massachusetts, United States) measuring at 520 nm. The NPs were imaged using a Talos F200 SEM with a 200-kV beam voltage (Fig. S1 in the Supplementary Material). Particle size distribution of 138.3±0.4  nm and concentration were independently evaluated using nanoparticle tracking analysis on a NanoSight NS300 system (Malvern Panalytical, Worcestershire, United Kingdom) (Fig. S2a in the Supplementary Material).

### 2.2 Animal Experiments

Eight-month-old and 10-month-old female Balb/c mice (Charles River Laboratories, Wilmington, Massachusetts, United States) were used for the NP biodistribution and imaging studies. All animal procedures were reviewed and approved by the University’s Institutional Animal Care and Use Committee (IACUC #20847) and adhered to the established standards and guidelines for the humane care of laboratory animals.

### 2.3 Animal Injections

**Fig. 1:**
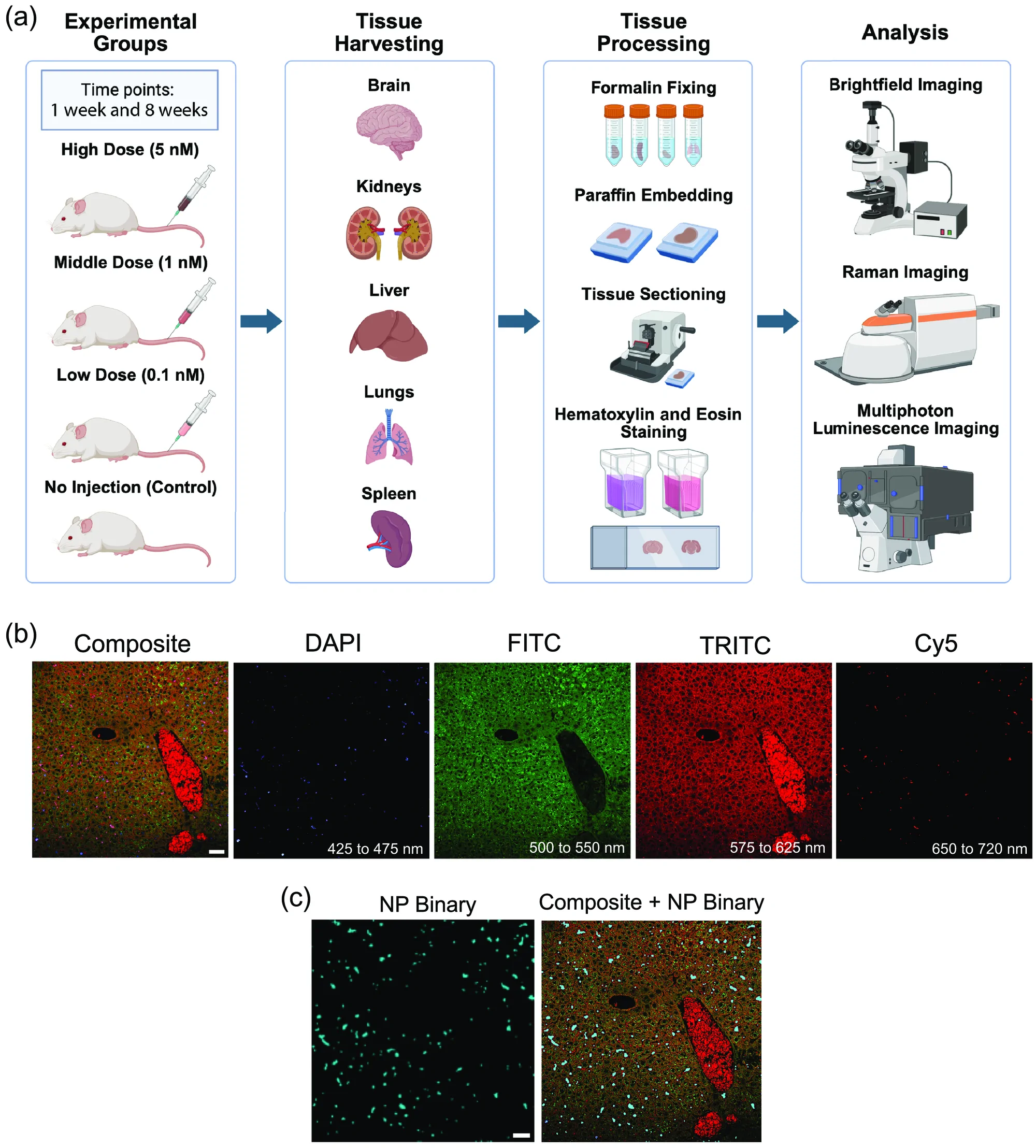
Experimental workflow and multimodal detection strategy for SERS AuNP imaging. (a) Schematic of the experimental design, including NP dosing (high, medium, low, and control), organ collection (brain, kidney, liver, lung, and spleen), tissue processing (fixation, embedding, sectioning, and H&E staining), and imaging using BF, Raman, and MPL microscopy. (b) MPL multi-channel images of H&E-stained tissue sections, including DAPI (425 to 475 nm), FITC (500 to 550 nm), TRITC (575 to 625 nm), and Cy5 (650 to 720 nm) emission channels, along with the corresponding composite image. Tissue structure is primarily observed through endogenous autofluorescence, while NP-associated emission is detected in specific channels (scale bars: 20  μm). (c) NP localization obtained by correlating DAPI and Cy5 channels to generate a binary NP detection mask and corresponding overlay on the MPL composite image to visualize NP distribution relative to tissue architecture (scale bars: 20  μm).

Animals were assigned to four experimental groups corresponding to 1- and 8-week post-injection time points to evaluate dose-dependent biodistribution of SERS AuNPs following short- and long-term exposure [Fig. 1(a)]. At the 1-week time point, one mouse received each SERS AuNP concentration of 5, 1, or 0.1 nM. The same experimental design was used at the 8-week time point, with one mouse receiving each concentration. Thus, each dose group included two mice in total, with one evaluated at 1 week and one evaluated at 8 weeks (5 nM, n=2; 1 nM, n=2; and 0.1 nM, n=2). One uninjected mouse served as the control (0 nM; n=1). Silica-coated SERS AuNPs were administered via IV injection using a butterfly needle at a total injection volume of 200  μL per animal. Lower-dose formulations (1 and 0.1 nM) were prepared by serial dilution of the high-dose suspension in 10 mM MOPS buffer (pH 7.4). Animals that successfully received injections were uniquely identified by tail markings for group tracking throughout the study.

### 2.4 Organ Harvesting, Processing, and Staining

After the designated post-injection time points, animals were euthanized under deep isoflurane anesthesia followed by cervical dislocation. Organs, including the liver, spleen, lungs, kidneys, and brain, were excised and gently rinsed with Phosphate-Buffered Saline (PBS) to remove residual blood. To facilitate fixation and downstream processing, tissues were prepared as follows: the liver was separated into individual lobes, with the right medial lobe bisected; the lungs were divided into left and right portions; the brain was separated into two hemispheres; the kidneys were collected individually and bisected along the coronal plane; and the spleen was collected intact without division. All tissues were then placed into 50-mL conical tubes containing 10% neutral buffered formalin (NBF) for fixation. Samples were maintained at 4°C for ∼48  h, with fresh 10% NBF replaced every 24 h to ensure adequate fixation based on tissue size and thickness. The samples were then transferred to 70% ethanol for storage. For each animal, three histological sections per organ were collected and analyzed as section-level replicates. All histological processing steps, including paraffin embedding, sectioning, and H&E staining, were performed by the Translational Pathology Core Facility at the USC Norris Comprehensive Cancer Center.

### 2.5 Brightfield, Raman, and MPL Imaging

Brightfield (BF) imaging was performed using a Leica MICA automated microscopy platform (Leica Microsystems, Inc., Deerfield, Illinois, United States) to assess the overall tissue morphology and histological features. A 1.6× objective lens was used to capture whole-organ sections, while a 20× objective lens was used to capture selected ROIs. Tiled image acquisition was used to generate stitched images for comprehensive visualization.

Raman imaging of the samples was conducted using an RA816 Biological Analyzer Raman microscope (Renishaw plc). The system was equipped with a 120-mW near-infrared semiconductor diode laser operating at a wavelength of 785 nm and a coverslip-corrected 40× objective lens with an NA of 0.50. Laser power was controlled using neutral density filtering, and spectral calibration was performed prior to imaging using a silicon reference standard. Raman maps were acquired in StreamLine imaging mode with sub-second integration times per pixel to generate spatially resolved spectral images. Whole-organ Raman scans were acquired using an exposure time of 0.261 s per pixel with a spatial step size of 54.3  μm and a binning factor of 41. ROI scans were collected using an exposure time of 0.415 s per pixel with a step size of 1.3  μm and no pixel binning. Laser power was reduced to 20% for whole-organ liver and spleen imaging; 80% for whole-organ brain, lung, and kidney imaging; and 50% for all ROI measurements. Raman ROI scans were acquired using a 600×600 acquisition grid with an acquisition time of ∼45  min per ROI. Whole-organ Raman scans required ∼45 to 60 min depending on organ size. The comparable acquisition times reflect the higher spatial sampling density used for ROI imaging relative to the coarser sampling used for whole-organ mapping.

MPL imaging was performed using a Nikon A1R HD upright multiphoton microscope (Nikon Instruments Inc., Melville, New York, United States) equipped with a Chameleon Discovery ultrafast tunable laser (Coherent Corp., Saxonburg, Pennsylvania, United States). Excitation was performed at 1040 nm, where 1% laser power corresponds to ∼100  mW at the sample. A Plan Apo 10× objective lens with an NA=0.45 was used for whole-organ and ROI imaging. Emission signals were collected through four detection channels corresponding to standard fluorescence bandpass filters: 450/50  nm [4′,6-diamidino-2-phenylindole (DAPI)], 525/50  nm [fluorescein isothiocyanate (FITC)], 600/50  nm [tetramethylrhodamine isothiocyanate (TRITC)], and 685/70  nm [cyanine 5 (Cy5)]. This multi-channel detection strategy enabled separation of broadband AuNP luminescence from tissue autofluorescence, with AuNP signal primarily detected in the DAPI and Cy5 channels. Laser power was set to 9% for liver and spleen imaging and 16% for brain, lung, and kidney imaging. MPL ROI images were acquired at 1024×1024  pixels (1.23  μm/pixel) with an acquisition time of ∼10  s per ROI. Whole-organ MPL images were acquired using tiled imaging at the same spatial sampling, with acquisition times of ∼30 to 50 min depending on organ size.

### 2.6 Imaging Quantitation and Analysis

Raman spectral analysis and image quantification were performed using the Renishaw WiRE software. Reference spectra were first acquired from pure SERS AuNP solutions prior to tissue imaging. These reference spectra were used to enable identification and quantification of SERS AuNP-associated signals within biological samples. Raman images were generated using a non-negative least squares fitting approach, in which each measured spectrum was decomposed into contributions from the reference spectra. The resulting weight values represent the relative contribution of the SERS signal at each pixel and were used to generate spatially resolved maps of NP distribution. Subsequent image processing and quantification were performed using ImageJ. All quantified values were used for downstream statistical analysis and comparison across experimental conditions.

MPL quantification was performed using the Nikon NIS-Elements software (version 5.30.06). Images were acquired across four emission channels (DAPI, FITC, TRITC, and Cy5), with NP identification based on correlated emission between the DAPI and Cy5 channels [Fig. 1(b)]. A spatial–spectral correlation approach was used to isolate AuNP signal from tissue autofluorescence. Raw MPL image stacks were first preprocessed to extract the DAPI and Cy5 channels, which exhibit strong AuNP luminescence and reduced background autofluorescence. For each pixel, a local Pearson correlation coefficient (r) was computed over a 5×5  pixel neighborhood between the DAPI and Cy5 channels, generating a floating point correlation map (range: 0 to 1). High correlation values indicate spatially co-localized emission across both channels, consistent with the broadband luminescence characteristics of AuNPs, while endogenous tissue signals are typically confined to a single channel. The resulting correlation map was thresholded (typically r>0.6 to 0.8) to generate a binary segmentation mask corresponding to NP-positive regions. The resulting binary masks were used to quantify SERS AuNP signal, including positive area fraction and aggregate size metrics [Fig. 1(c)].

### 2.7 Statistical Analysis

Quantitative data are presented as mean ± standard error of the mean (SEM) unless otherwise noted. The measurements were obtained from three tissue sections per organ from one mouse for each experimental condition. Accordingly, section-level measurements represent technical replicates and not independent biological replicates. Correlation analyses between Raman and MPL measurements were performed using linear regression, Pearson correlation, and Spearman’s rank correlation, depending on the distribution and type of comparison. Statistical comparisons were analyzed using Student’s t tests. Statistical significance was defined at a 95% confidence interval (p<0.05). Statistical significance is indicated in the figures as ns (not significant), *(p<0.05), **(p<0.01), ***(p<0.001), and ****(p<0.0001).

## 3 Results

### 3.1 Multimodal Detection of SERS AuNPs in Tissue Sections

BF microscopy is widely used in histological analysis to evaluate tissue morphology and pathological features, particularly in H&E-stained sections.^20^^,^^54^ Its accessibility and ability to provide structural context have led to its use in NP biodistribution studies to assess tissue-level changes and, in some cases, to infer NP accumulation.^56^ However, BF imaging does not provide NP-specific contrast as the NPs are below the diffraction limit and ultimately relies on large NP aggregates that present as dark pigmented clusters, which may limit its sensitivity when NPs are present at low concentrations or are diffusely distributed.

**Fig. 2:**
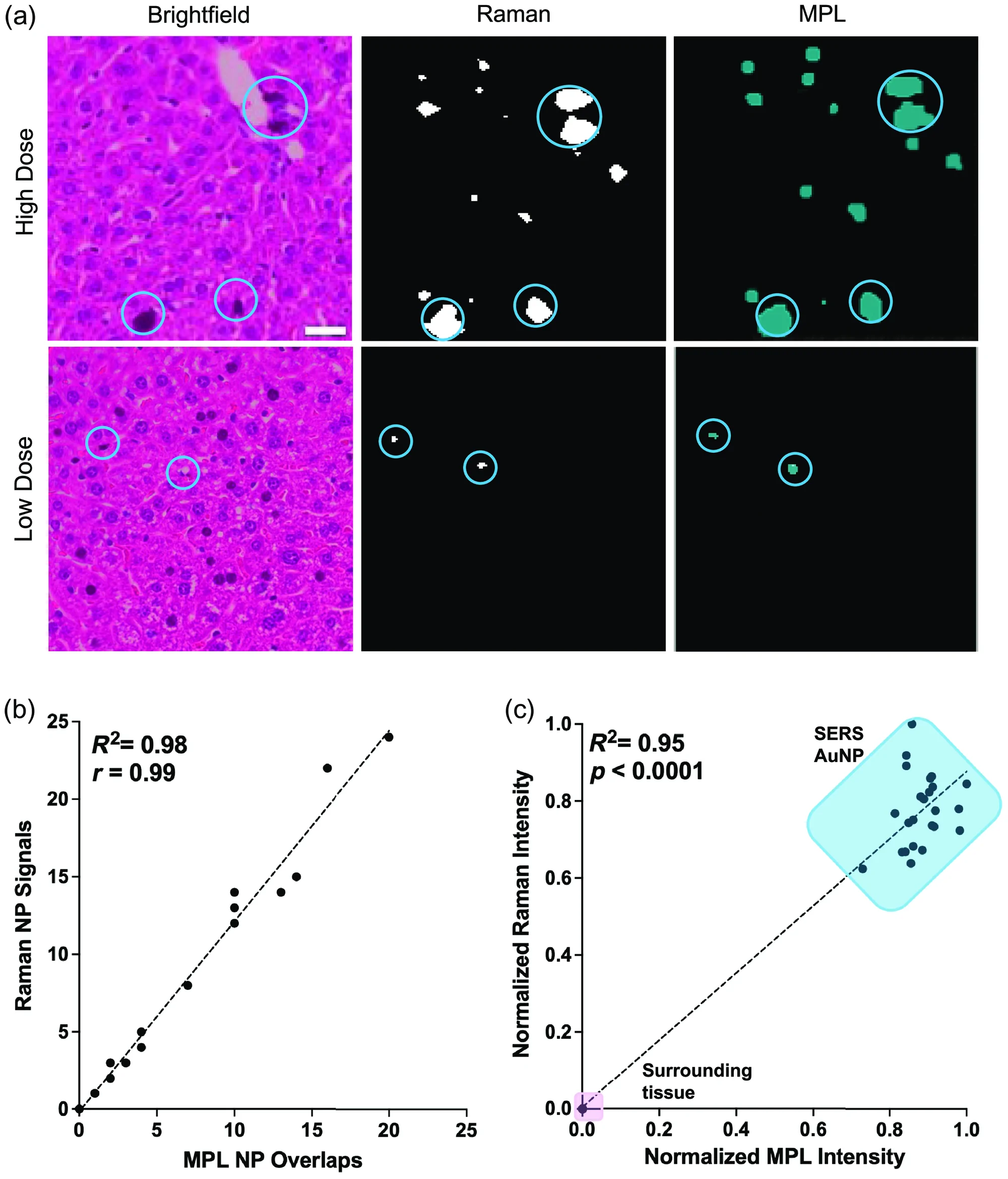
Multimodal imaging of SERS AuNP detection in liver tissue at different concentrations. (a) BF images of liver sections following administration of SERS AuNPs at high (5 nM) and low (0.1 nM) concentrations. NP aggregates are visible in BF at the high concentration (circled) but are not as visible at the low concentration. Raman images show NP-associated signal at both concentrations. MPL correlation binary layer (DAPI + Cy5) highlights localized NP emission. Circled regions indicate representative NP-positive areas identified across imaging modalities. Raman and MPL imaging detect NP-associated signals at both concentrations, whereas BF imaging shows no apparent contrast at low concentration (scale bar: 20  μm). (b) Correlation analysis of Raman- and MPL-detected NP-positive regions across corresponding ROIs. NP-positive regions were identified from Raman images, and these regions were applied to the corresponding MPL images to determine whether signal was also detected within the same regions. The axes represent the number of corresponding NP-positive regions identified within each ROI. Strong agreement among modalities was observed ($R^{2}=0.98$, r=0.99), indicating consistent identification of SERS AuNP-associated regions by MPL imaging. (c) Correlation of normalized Raman and MPL signal intensities from individual NP-positive regions and surrounding tissue background within a representative liver ROI demonstrated strong agreement among modalities ($R^{2}=0.95$, p<0.0001), with MPL signal corresponding to Raman-detected SERS AuNP signal.

To examine these limitations, liver tissue sections from mice administered SERS AuNPs IV at different concentrations were imaged using BF, Raman, and MPL microscopy [Fig. 2(a)]. The liver was selected due to its known role in NP clearance and accumulation. At the higher concentration (5 nM), BF images showed dark pigmented features that are consistent with regions of increased NP accumulation. These features appeared as punctate structures distributed within the tissue and were more readily identifiable in areas with higher signal density. At the lower concentration (0.1 nM), BF images of tissues lacked comparable features and visual contrast, so SERS AuNPs could not be distinguished from surrounding tissue structures.

Raman and MPL imaging were used to assess SERS AuNP signal within the same sections. Raman imaging detected SERS AuNPs through characteristic vibrational signatures associated with the BPE reporter molecule (Fig. S2b in the Supplementary Material). MPL imaging was used to identify luminescence originating from the gold core under multiphoton excitation. AuNP luminescence is most distinct in the DAPI and Cy5 emission ranges relative to endogenous tissue signal.^24^ Based on this spectral separation, a binary NP detection map was generated by correlating the DAPI and Cy5 channels, enabling selective identification of spatially co-localized emission while suppressing background autofluorescence [Fig. 2(a) MPL images]. Notably, at the lower concentration, where BF imaging did not reveal NP features, both Raman and MPL modalities were still able to detect localized signals. To quantitatively evaluate this spatial correspondence, NP-positive regions identified from multiple Raman ROIs collected across organs were analyzed and compared against the corresponding MPL ROIs [Fig. 2(b)]. Raman-identified NP-positive regions were saved and applied to the corresponding MPL images to determine whether signal was also detected within the same regions. Linear regression demonstrated strong agreement between Raman- and MPL-detected NP-positive regions ($R^{2}=0.98$), with a high Pearson correlation coefficient (r=0.99), indicating that regions classified as NP-positive in Raman imaging were consistently detected by MPL imaging.

To further assess local signal agreement among modalities at the local tissue level, intensity was measured from individual NP-positive regions and surrounding background tissue within liver ROIs [Fig. 2(c)]. Normalized MPL and Raman intensity measurements exhibited strong positive correlation ($R^{2}=0.95$), demonstrating that regions with elevated MPL signal corresponded to regions with increased Raman signal intensity. Together, these findings indicate that Raman and MPL imaging provide highly correlated detection of SERS AuNP-associated signal and offer increased sensitivity for nanoparticle detection in tissue sections, including conditions where such features are not readily apparent in BF imaging.

By utilizing a non-destructive imaging workflow, we were able to perform all three imaging modalities on the identical H&E-stained section. This approach avoids the use of serial sections and enables multiple imaging sessions to evaluate the precise localization of AuNPs and any neighboring morphological variations across the same tissue architecture.

### 3.2 Dose-Dependent Quantification and Cross-Modal Validation

To further evaluate the sensitivity and quantitative performance of MPL imaging, dose-dependent changes in SERS AuNP distribution were assessed in liver and spleen tissues across multiple administered concentrations. These organs were selected based on their established roles in NP uptake and clearance, where accumulation is expected to vary with dose.

**Fig. 3:**
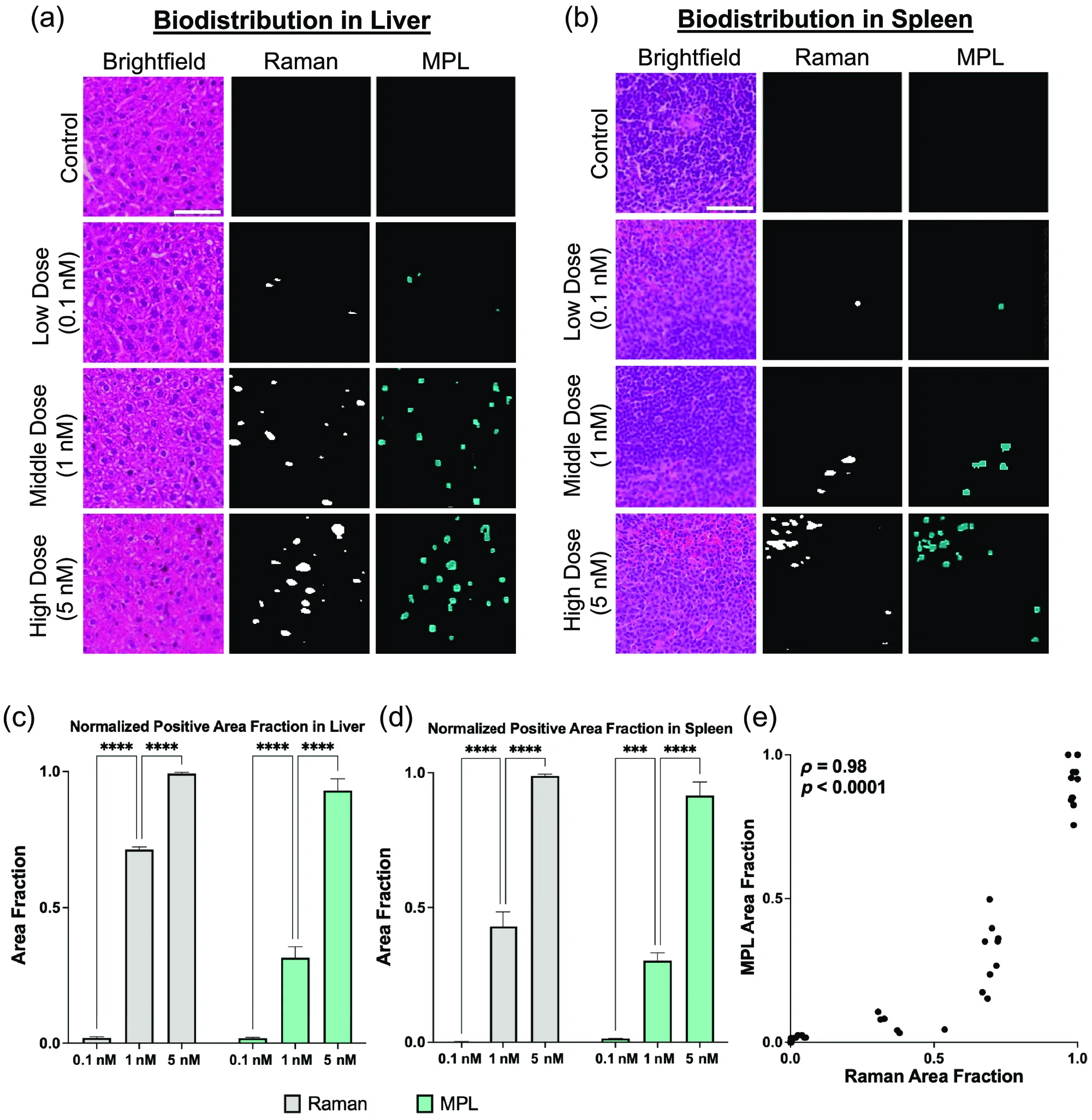
Dose-dependent detection of SERS AuNPs in the liver and spleen using Raman and MPL imaging. (a) Representative BF, Raman, and MPL images of liver tissue ROIs acquired from mice administered SERS AuNPs at increasing concentrations (0, 0.1, 1, and 5 nM). NP-associated signal is not apparent in BF at low concentrations but is detected by both Raman and MPL imaging, with increasing signal intensity observed with dose. (b) Representative BF, Raman, and MPL images of spleen ROIs at corresponding concentrations, showing a similar dose-dependent increase in NP-associated signal. (c) Quantification of normalized positive pixel area fraction from whole-organ liver scans for Raman and MPL (n=3 whole-organ scans per mouse), showing an increase in nanoparticle-associated signal with increasing dose for both modalities. Positive pixels were defined as those exceeding a predefined signal threshold, and area fraction was calculated as the proportion of positive pixels relative to the total tissue area. (d) Quantification of normalized positive area fraction from whole-organ spleen scans for Raman and MPL (n=3 whole-organ scans per mouse), demonstrating consistent dose-dependent trends. (e) Spearman’s rank correlation between Raman- and MPL-positive pixel area fraction measurements across all conditions and all collected organs, indicating agreement between the two modalities in detecting SERS AuNP signal. Statistical significance is denoted as ***p<0.001 and ****p<0.0001 (scale bar: 50  μm).

Representative ROI images from liver tissue [Fig. 3(a)] show a progressive increase in signal with increasing administered concentration for both Raman and MPL modalities. At the lowest concentration (0.1 nM), signal is sparse and appears as isolated, localized regions distributed throughout the tissue. At medium (1 nM) and higher concentrations (5 nM), signal becomes increasingly widespread, with greater spatial occupancy and higher density of NP-positive regions. This transition from localized to more continuous signal distribution is observed for both imaging modalities. In comparison, BF imaging does not consistently reveal corresponding NP features at lower concentrations, and contrast remains dominated by underlying tissue morphology. A similar dose-dependent trend is observed in spleen tissue [Fig. 3(b)]. At low concentration, signal appears limited to discrete regions, while increasing concentration results in broader spatial distribution and higher overall occupancy. The spleen exhibits a more heterogeneous pattern of signal distribution relative to the liver, particularly at intermediate concentrations, which may reflect differences in tissue architecture and NP accumulation pathways. Both Raman and MPL modalities capture consistent increases in SERS AuNP signal with an increase in dose concentration.

To quantify these observations, whole-organ scans were analyzed to determine the normalized positive area fraction for each modality. In liver tissue [Fig. 3(c)], both Raman and MPL measurements exhibited a monotonic increase in signal with increasing dose. For Raman, values increased from 0.019 to 0.714 to 0.993 across higher concentrations. MPL measurements followed a similar trend, increasing from 0.019 to 0.316 to 0.930. At the lowest concentration, signal was minimal, while intermediate and high concentrations showed substantial increases in NP-positive area, reflecting broader spatial distribution within the tissue. Although MPL values appeared lower at intermediate concentration, both modalities approached similar levels at the highest dose, where signal became more uniformly distributed. In spleen tissue [Fig. 3(d)], a similar dose-dependent trend was observed. Raman measurements increased from 0.002 to 0.430 to 0.988, while MPL measurements increased from 0.014 to 0.303 to 0.915, respectively. Signal at lower concentrations remained limited, while higher concentrations resulted in greater occupancy of SERS AuNP signal. Compared with the liver, the spleen exhibited greater variability at lower and intermediate concentrations, consistent with the more heterogeneous spatial patterns observed in the representative images.

To assess agreement among modalities, a correlation analysis was performed across all organs and dose conditions [Fig. 3(e)]. Spearman correlation (non-parametric correlation) was calculated using measurements from multiple whole-organ scans spanning five organs and three administered doses. MPL and Raman measurements exhibited a strong positive correlation (Spearman’s ρ=0.9768, p<0.0001), indicating that increases in MPL signal correspond to increases in Raman-detected SERS AuNP signal. This relationship was maintained across both liver and spleen tissues and across the full range of administered concentrations, despite the differences in absolute signal magnitude between the two modalities.

### 3.3 Whole-Organ Biodistribution of SERS AuNPs

To further evaluate the spatial distribution of SERS AuNPs across the body, we assessed how NP signal varies across organs (liver, spleen, lung, kidney, and brain) with distinct physiological roles in NP uptake, filtration, and clearance and compared the consistency of Raman and MPL measurements at the organ level. Representative images were obtained from a 1-week post-injection time point at a 5-nM dose, where NP signal was detectable across all organs, enabling direct comparison of biodistribution patterns.

**Fig. 4:**
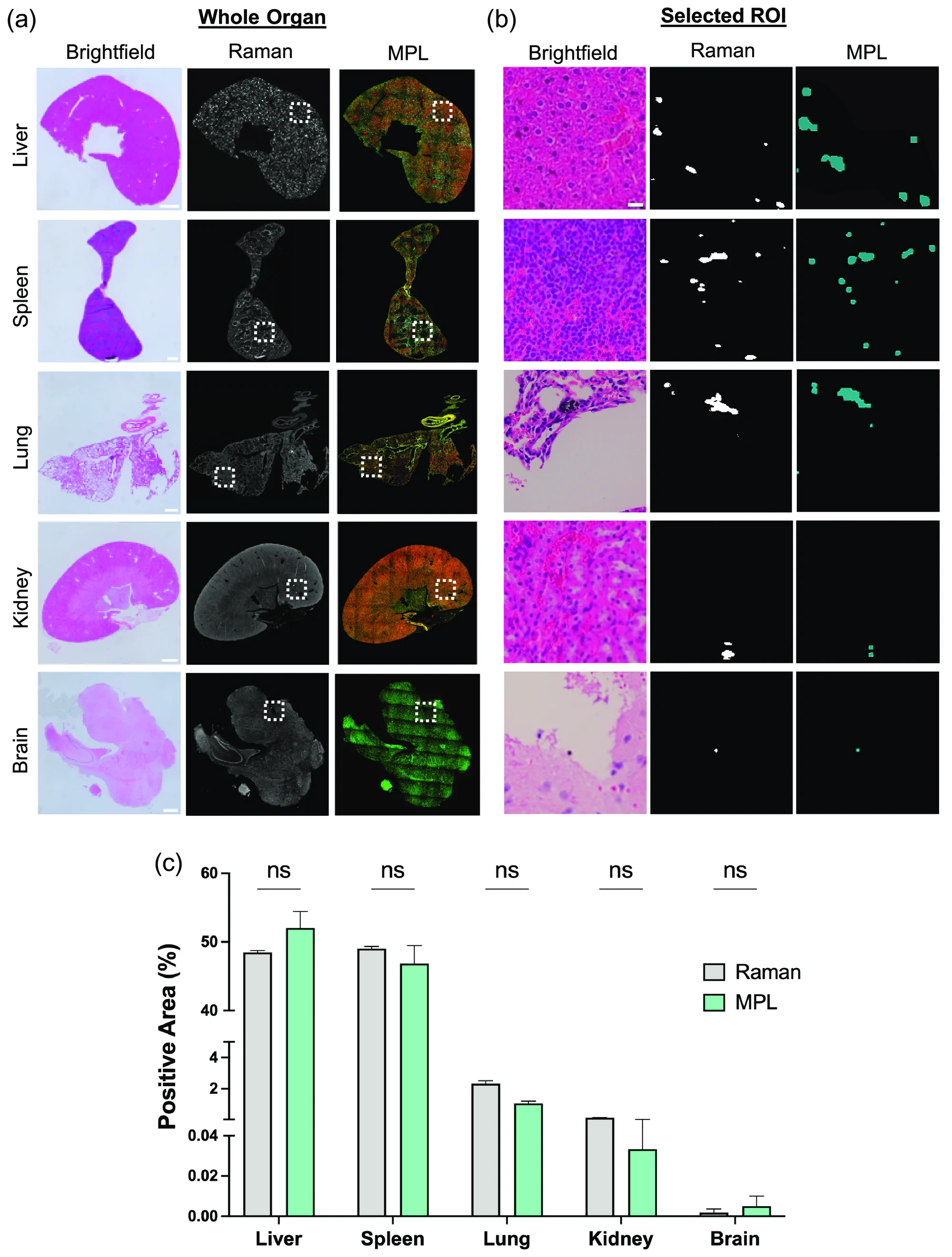
Whole-organ and specific ROI biodistributions of SERS AuNPs across major organs. (a) Representative whole-organ BF, Raman, and MPL images of the liver, spleen, lung, kidney, and brain collected after 1 week following administration of SERS AuNPs. White dashed boxes indicate ROIs selected for higher-magnification analysis (scale bar: 1 mm). (b) Corresponding ROI-level BF, Raman, and MPL images are shown to illustrate localized NP-associated signal within each organ (scale bar: 20  μm). (c) Normalized positive area fraction across organs quantified from whole-organ Raman and MPL scans, with signal in each organ expressed as a percentage of the total detected signal across all five organs. Both modalities demonstrated similar biodistribution patterns, with higher NP signal observed in the liver and spleen relative to the lung, kidney, and brain. No significant differences in normalized positive area fraction were observed between Raman and MPL measurements within each organ (ns). Data are presented as mean ± SEM.

Whole-organ images [Fig. 4(a)] demonstrate clear differences in NP signal across organs. In both Raman and MPL images, the highest signal intensity and occupancy were observed in the liver and spleen, while substantially lower signal levels were detected in lung, kidney, and brain tissues. These trends were consistent across both imaging modalities. BF images provided structural context for each organ but did not reveal NP features, particularly in tissues with low NP accumulation. Selected ROI images [Fig. 4(b)] further illustrate the localized distribution of NP signal within each organ. In the liver and spleen, NP signal appeared relatively widespread, with multiple regions of accumulation observed throughout the tissue. In lung tissue, signal was more localized and appeared in discrete clusters, often associated with specific structural features. Kidney and brain tissues exhibited minimal detectable signal in both modalities, with only sparse NP regions observed.

To quantify these observations, the normalized positive area fraction was calculated for each organ based on whole-organ scans [Fig. 4(c)]. Signal in each organ was expressed as a fraction of the total NP-positive area detected across all five organs, enabling relative comparison of biodistribution patterns among modalities. For Raman measurements, the distribution across the liver, spleen, lung, kidney, and brain was 48.49%, 49.02%, 2.33%, 0.15%, and 0.002%, respectively. MPL measurements followed a similar distribution pattern, with values of 52.04%, 46.87%, 1.06%, 0.03%, and 0.005%, respectively. Both modalities demonstrated comparable organ-level biodistribution profiles, with the majority of NP signal localized within the liver and spleen and lower signal observed in lung, kidney, and brain tissue. No statistically significant differences were observed between Raman and MPL measurements within each organ (ns), indicating strong agreement among modalities across multiple tissue types. These findings support the use of MPL as a label-free approach for assessing nanoparticle distribution at the organ level, while Raman imaging provides complementary SERS NP spectral specificity.

### 3.4 Time-Dependent Redistribution and NP Aggregation

To evaluate how NP signal and spatial organization change over time and to compare the consistency of these changes across Raman and MPL modalities, imaging was performed at 1- and 8-week time points following IV administration.

**Fig. 5:**
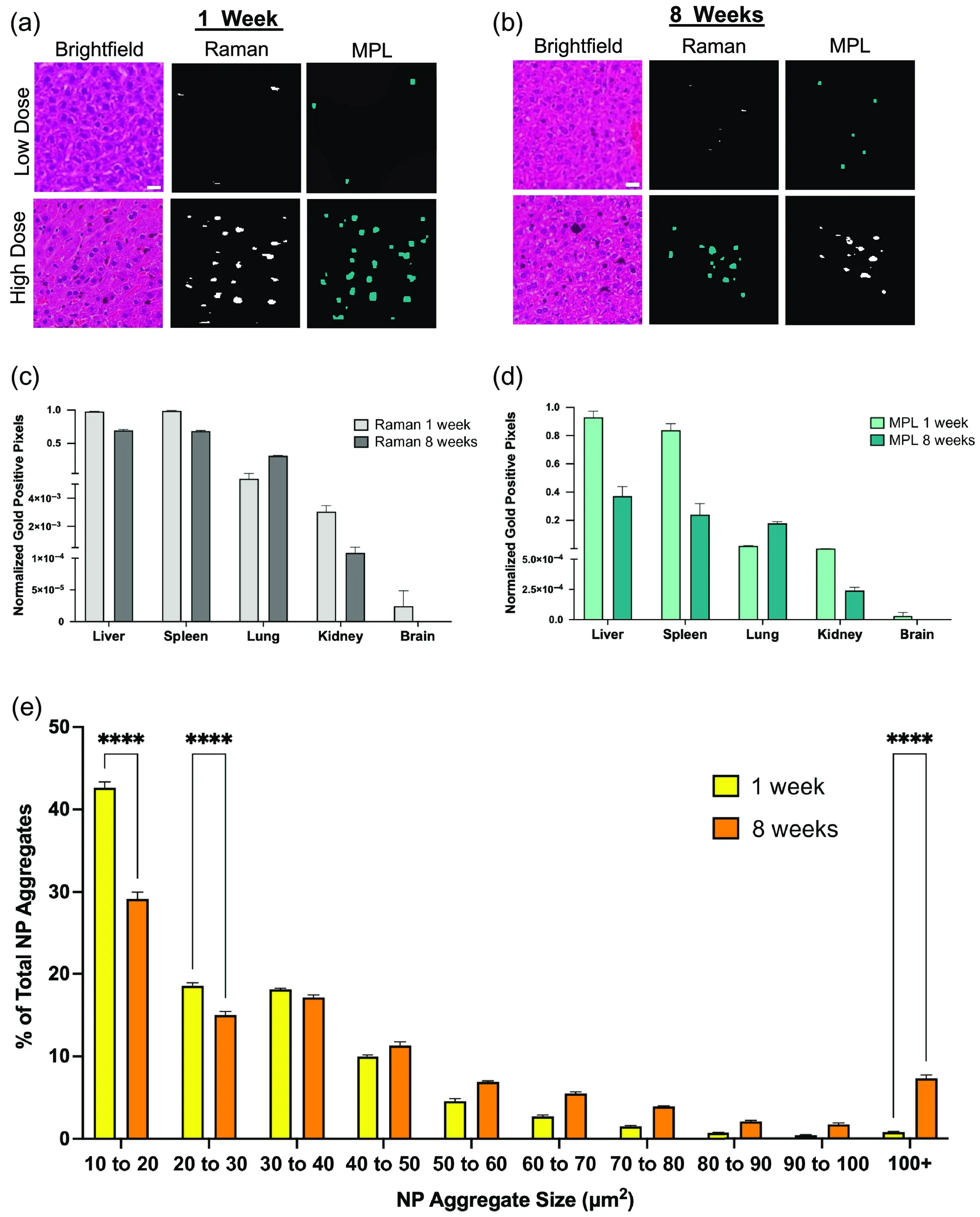
Time-dependent changes in SERS AuNP biodistribution and aggregation. (a) BF, Raman, and MPL images of liver tissue ROIs at 1 week following the administration of SERS AuNPs at low (0.1 nM) and high (5 nM) concentrations (scale bar: 20   μm). (b) Corresponding BF, Raman, and MPL images of liver ROIs at 8-week post-administration, showing differences in NP-associated signal distribution compared with the 1-week time point (scale bar: 20  μm). (c) Quantification of normalized positive area fraction across organs (liver, spleen, lung, kidney, and brain) from whole-organ Raman scans at 1 and 8 weeks. Data were normalized to the maximum positive area fraction measured and presented as mean ± SEM. (d) Corresponding quantification from MPL measurements, showing comparable trends across time points. (e) Distribution of NP aggregate sizes at 1 and 8 weeks, indicating a shift toward larger aggregate sizes at the later time point (8 weeks). Aggregate size is reported as area ($\mu m^{2}$), with values expressed as the percentage of total detected NP aggregates. Statistical significance is denoted as ****p<0.0001.

Representative ROI images from liver tissue at 1 week [Fig. 5(a)] and 8 weeks [Fig. 5(b)] demonstrate observable differences in NP signal distribution. At both time points, Raman and MPL imaging detect NP signal at both low (0.1 nM) and high (5 nM) doses, while BF imaging continues to provide structural context without clear NP-specific contrast at lower concentrations. At the high dose, signal at 1 week appeared more dispersed, with numerous smaller, spatially separated NP-positive regions distributed throughout the tissue. At 8 weeks, the spatial distribution appeared more localized, with fewer but more clustered regions of signal. This qualitative shift suggests a transition in NP organization over time. A similar trend is observed in both Raman and MPL images, with consistent spatial patterns across modalities.

Normalized positive area fraction across organs for both time points using Raman [Fig. 5(c)] shows that at 1 week, the distribution across the liver, spleen, lung, kidney, and brain was 0.9773, 0.9880, 0.0470, 0.0031, and 0.00002, respectively. At 8 weeks, this distribution shifted to 0.6963, 0.6857, 0.3153, 0.0004, and 0, respectively. These results suggest that although the liver and spleen remain the primary sites of NP signal, their relative contribution decreases over time, accompanied by an increase in signal detected in lung tissue. Corresponding MPL quantification [Fig. 5(d)] demonstrated a similar redistribution pattern. At 1 week, the distribution across the liver, spleen, lung, kidney, and brain was 0.9303, 0.8379, 0.0190, 0.0005, and 0.00003, respectively, while at 8 weeks, this shifted to 0.3714, 0.2409, 0.1790, 0.0002, and 0.0000, respectively. Consistent with Raman measurements, MPL data show a decrease in relative signal in the liver and spleen and an increase in lung-associated signal over time, while the kidney and brain remain minimally represented. Despite the differences in absolute magnitude among modalities, the overall trends remain in agreement.

Aggregate size analysis was performed using MPL-derived binary segmentation masks, which enable direct spatial quantification of NP-positive regions. This approach leverages the correlation-based detection of AuNP signal between the DAPI and Cy5 channels, producing well-defined boundaries suitable for object-based analysis. SERS AuNP aggregate size distribution [Fig. 5(e)] shows that at 1 week, NP signal was predominantly distributed across smaller aggregate size ranges, with the highest proportion observed in the 10 to ${20\;\mu m}^{2}$ bin, which decreased from 0.4264 to 0.2916 at 8 weeks. Similar decreases were observed in the 20 to ${30\;\mu m}^{2}$ and 30 to ${40\;\mu m}^{2}$ bins, which declined from 0.1855 to 0.1501 and from 0.1812 to 0.1714, respectively. In contrast, larger aggregate size bins exhibited increased contributions over time, including 50 to $60\;{\mu m}^{2}$ (increasing from 0.0457 to 0.0691), 70 to $80\;{\mu m}^{2}$ (from 0.0151 to 0.0391), and 80 to $90\;{\mu m}^{2}$ (from 0.0073 to 0.0207). Notably, the largest aggregate category ($>100\;{\mu m}^{2}$) increased from 0.0081 to 0.0730. These shifts indicate a redistribution toward larger aggregate sizes over time, accompanied by a reduction in smaller aggregates.

These results suggest that NP signal becomes more spatially confined at later time points. Rather than remaining uniformly dispersed across the tissues, NPs appear to undergo spatial condensation into fewer, more localized regions of accumulation, with an increased tendency toward aggregation which has been previously observed and reported.^24^ This trend is consistently observed across both Raman and MPL modalities.

## 4 Discussion

This study establishes a multimodal imaging framework combining MPL and Raman microscopy to evaluate the biodistribution and evolution of SERS AuNPs in biological systems on the subcellular scale. The results demonstrate that MPL enables label-free detection of NP-associated signal with high spatial resolution, while Raman imaging provides spectral specificity through detection of the SERS reporter. Together, these modalities provide complementary information, enabling both identification and localization of SERS AuNPs across multiple spatial scales.

We observed strong agreement between MPL and Raman measurements across doses, organs, and time points. Spatial overlap between Raman and MPL NP-positive regions demonstrated high correspondence among modalities, with ROI-based analysis showing strong linear agreement ($R^{2}=0.98$) and high Pearson correlation (r=0.99). In addition, normalized Raman and MPL signal intensities extracted from corresponding nanoparticle-positive regions exhibited strong correlation ($R^{2}=0.95$), indicating that regions with elevated MPL signal correspond closely to Raman-confirmed SERS AuNP signal. Differences in the quantitative values between Raman and MPL measurements are expected and reflect fundamental differences in imaging modality, spatial sampling, and signal generation. Raman imaging detects SERS AuNPs through highly distinct vibrational signatures generated by the organic Raman reporter coating the nanoparticle surface, providing strong chemical contrast and high detection specificity.^49^ In contrast, MPL imaging relies on intrinsic gold luminescence and correlation-based segmentation, providing more spatially localized signal detection based on the physical optical properties of the AuNP core. As a result, MPL-derived measurements may appear lower in magnitude, particularly at intermediate concentrations, as signal classification is more spatially constrained.

Whole-organ biodistribution analysis revealed that SERS AuNPs preferentially accumulate in the liver and spleen, consistent with their known roles in 140-nm NP clearance and uptake by the reticuloendothelial system. Both Raman and MPL imaging showed minimal signal in kidney and brain tissues. However, low levels of NP signal were detected in high-dose (5 nM) mouse brain tissue at the 1-week time point. The spatial distribution of this signal appeared localized rather than widespread, with patterns suggestive of association with vascular structures. This observation may indicate that NPs are present within larger blood vessels or regions of vascular retention rather than having penetrated broadly into the brain parenchyma, which is consistent with the restrictive nature of the blood–brain barrier. Further studies would be needed to determine the precise transport pathway and localization.

Longitudinal analysis further revealed time-dependent changes in NP distribution and organization. At 8 weeks, a reduction in normalized positive area fraction was observed in the liver and spleen, accompanied by an increase in signal detected in lung tissue. In parallel, aggregate size analysis demonstrated a shift toward larger NPs clusters over time, with a decrease in smaller aggregates and increased contribution from larger size bins. These results suggest that NPs undergo spatial reorganization following initial accumulation, transitioning from a more dispersed distribution to a more clustered and spatially confined state. Such aggregation behavior may reflect biological processes including macrophage cellular uptake, intracellular trafficking, or long-term retention within specific tissue compartments.^57^ MPL imaging provided higher resolution for NP detection, enabling more precise delineation of NP-positive regions compared with Raman imaging.

The nonlinear excitation mechanism of MPL confines signal generation to the focal volume and reduces background contributions, resulting in clearer spatial boundaries. This higher spatial resolution, combined with correlation-based segmentation, allows for robust object-based analysis of NP clusters. In contrast, Raman imaging produces spectrally derived intensity maps that are well-suited for identifying SERS NP presence but are less amenable to precise spatial segmentation. For this reason, MPL imaging was used for aggregate size quantification, as it provides the spatial information required for reliable measurement of NP aggregate distribution. Despite these advantages, MPL imaging has limitations. At lower NP concentrations, MPL signal can be more susceptible to background noise and variability, which may reduce contrast in sparsely distributed regions. In comparison, Raman imaging provides higher sensitivity and specificity through detection of unique SERS reporter Raman signals. These differences highlight the importance of a multimodal approach, where MPL provides spatially resolved, label-free imaging and Raman serves as a validation tool for confirming SERS NP identity.

Future studies could further extend this label-free multimodal imaging framework to evaluate longer post-injection time points and better understand the long-term fate and persistence of SERS AuNPs within biological tissues. The time-dependent changes in NP organization observed in this study suggest that NP distribution continues to evolve following initial accumulation, highlighting the importance of longitudinal analysis for understanding long-term biodistribution behavior. In addition to tracking NP localization, future work could incorporate histological and immunohistochemical analysis to evaluate potential biological responses associated with persistent NP retention, including tissue remodeling, fibrosis, inflammation, or apoptosis. Importantly, the non-destructive imaging workflow established in this study preserves tissue architecture and enables repeated multimodal imaging on the same H&E-prepared sections without compromising downstream histological evaluation. This provides a valuable platform for directly correlating nanoparticle localization with tissue morphology and pathological changes within the same tissue microenvironment. Lastly, this study demonstrates the strong potential of SERS AuNPs as multimodal imaging probes. The unique optical properties of SERS AuNPs, including intrinsic MPL signal and highly specific Raman signatures, support their future use as multifunctional *in vivo* contrast agents capable of enabling both spectroscopic validation and spatially resolved imaging across complex biological systems.

## 5 Conclusion

This work demonstrates that MPL can be used as a label-free approach to detect and localize SERS AuNPs in biological tissues, with Raman microscopy providing orthogonal validation through molecular specificity. The strong spatial agreement and correlation among modalities indicate that MPL-detected signals correspond to SERS AuNPs, supporting its use for spatially resolved NP imaging without the need for additional labels. Using this framework, we observed organ-dependent distribution consistent with established NP clearance pathways, as well as time-dependent changes in signal organization, including increased spatial confinement and aggregation at later time points. MPL enabled quantitative analysis of these spatial features, while Raman confirmed SERS AuNP identity across conditions.

Our study provides a multimodal strategy in which MPL enables high-resolution, label-free visualization while Raman imaging serves as a confirmatory tool for SERS AuNP identification. This approach addresses current limitations in nanoparticle biodistribution studies by enabling non-destructive, spatially resolved microscopic analysis over extended time scales. Furthermore, it offers a versatile strategy to evaluate how factors such as subject age, AuNP dosage, and different administration routes influence biodistribution dynamics. More broadly, the intrinsic optical properties of SERS AuNPs, including their high sensitivity and multiplexing capability, continue to demonstrate strong potential as multifunctional imaging contrast agents. By enabling detailed characterization of long-term nanoparticle distribution and retention within various biological tissues, this multimodal framework may further support future studies aimed at understanding the long-term biological effects of SERS AuNPs and facilitate their continued development toward clinical translation.

## Supplementary Material

10.1117/1.BIOS.3.4.043104.s01

## Data Availability

The data supporting the conclusions of this study are available from the corresponding author upon reasonable request.
