# Supplementary material for: Multimodal optical imaging strategy to evaluate the microscopic biodistribution of SERS gold nanoparticles across histology sections
Source: Biophotonics Discov. 2026 Sep 10;3(4):043104. doi: 10.1117/1.BIOS.3.4.043104 (PMC13561246; doi:10.1117/1.BIOS.3.4.043104)
Supplement: Supplementary file 1 [file BIOS_003_043104_SD001.pdf]

(a)

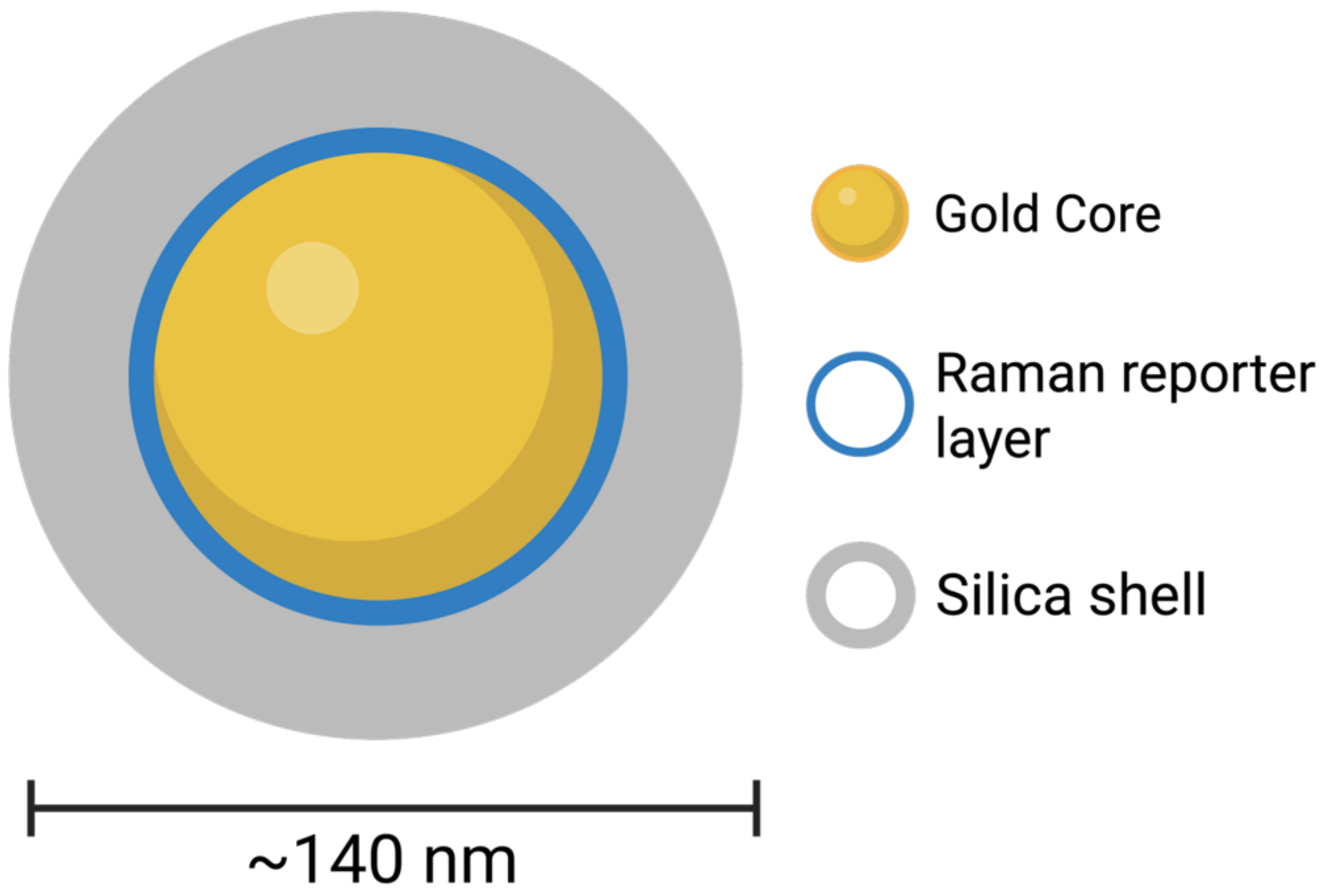

(b)

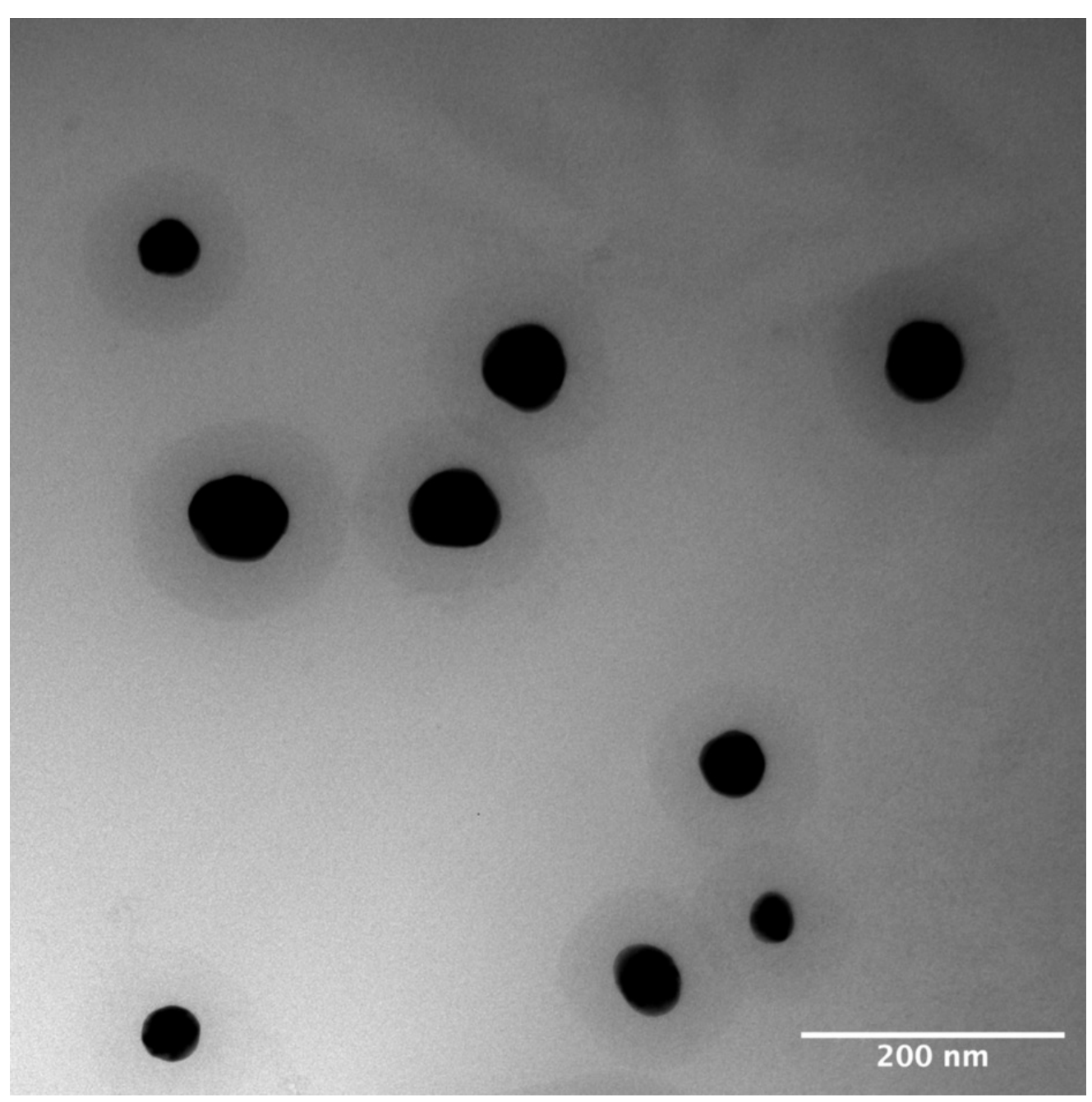

Figure S1. Structural and morphological characterization of SERS AuNPs.
(a) Schematic illustration of the SERS AuNP architecture, consisting of a gold core, Raman reporter layer, and silica shell, with an overall diameter of approximately ~140 nm. (b) Representative TEM image showing NP morphology and size distribution. Individual NPs appear as electron-dense spherical structures with relatively uniform size and minimal aggregation. (Scale bar: 200 nm)

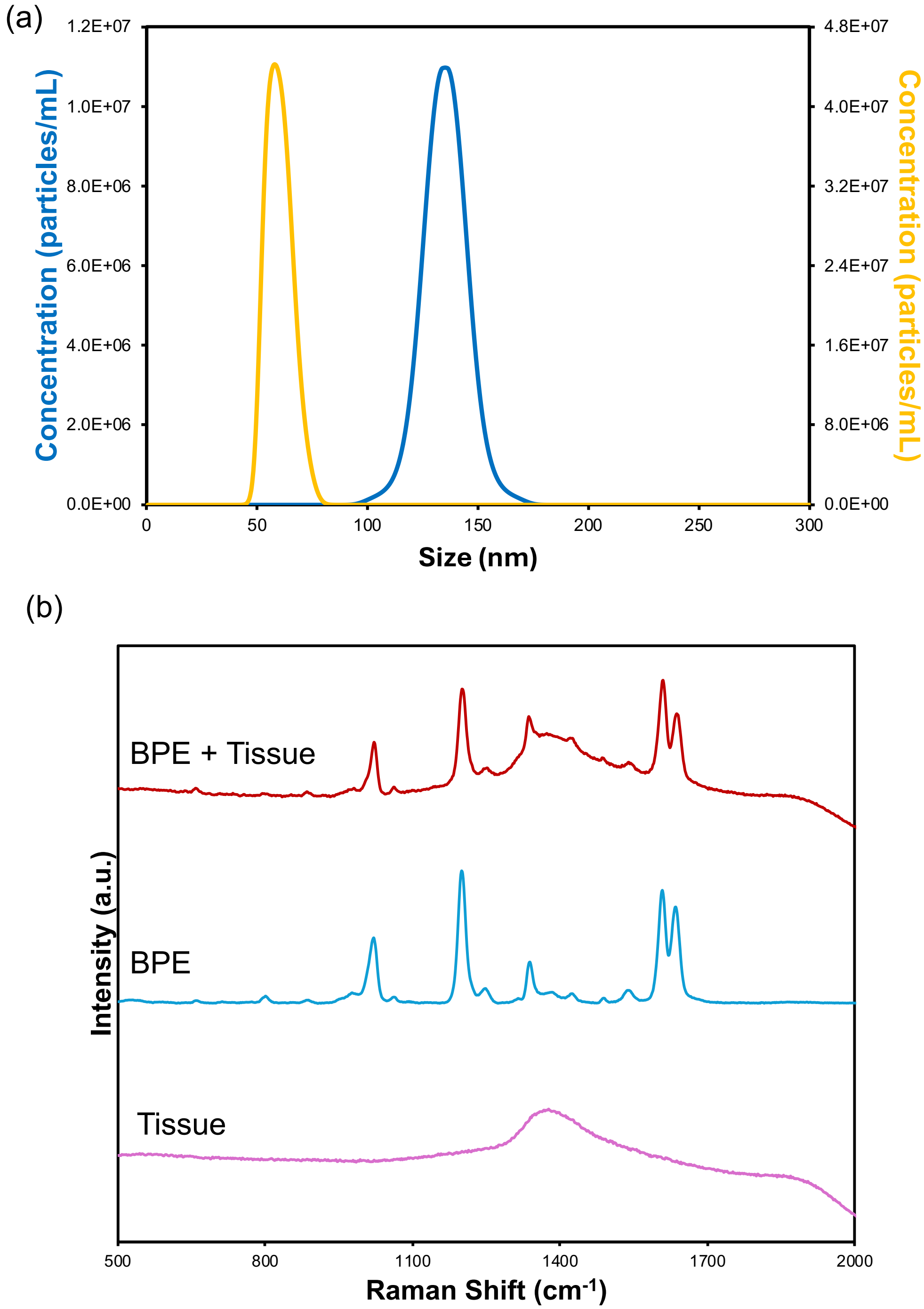

Figure S2. Spectral and size characterization of SERS AuNPs. (a) NP size distribution measured by particle sizing analysis, showing a dominant blue peak centered around ~140 nm corresponding to SERS AuNPs. The secondary distribution in yellow (~50 nm) represents the gold core prior to silica shell encapsulation. (b) Raman spectra of the BPE reporter, tissue background, and BPE in the presence of tissue, demonstrating distinct characteristic peaks of the reporter molecule that remain detectable in biological environments.
